# Supplementary material for: Adherence to High Dietary Diversity and Incident Cognitive Impairment for the Oldest-Old: A Community-Based, Nationwide Cohort Study
Source: Nutrients. 2022 Oct 27;14(21):4530. doi: 10.3390/nu14214530 (PMC9655345; doi:10.3390/nu14214530)
Supplement: Supplementary file 1 [file nutrients-14-04530-s001.zip › nutrients-1978728-supplementary.pdf]

Adherence to high dietary diversity and incident cognitive impairment for the oldest-old: a community-based, nationwide cohort study

Figure S1. Flow chart of the study population. CLHLS: Chinese Longitudinal Health Longevity Study 2

Table S1. The Chinese Version of the Mini-Mental State Exam (MMSE)[1]. 3

Table S2. The association between DDS change patterns and risk of cognitive impairment in subgroups (univariate model). 4

Table S3. β-Coefficients and 95%CI for the association of the DDS change patterns with MMSE score changes over Follow-up Time (N=6237): results from Linear Mixed-effects models 6

Table S4. Characteristics of 6237 participants at baseline 7

Table S5. Subgroup analysis for the association of DDS change with cognitive impairment ... 9

6.S-Sensitivity analyses 11

6.1 Excluded participants who lost to follow-up or died in the second 3 years 11

6.2 Using the definition of cognitive impairment (MMSE score less than 24) 13

6.3 Excluded participants with chronic diseases (hypertension, diabetes, cancer, cerebrovascular diseases) at baseline 15

6.4 Education was treated as a continuous variable 17

Figure S1. Flow chart of the study population. CLHLS: Chinese Longitudinal Health Longevity Study

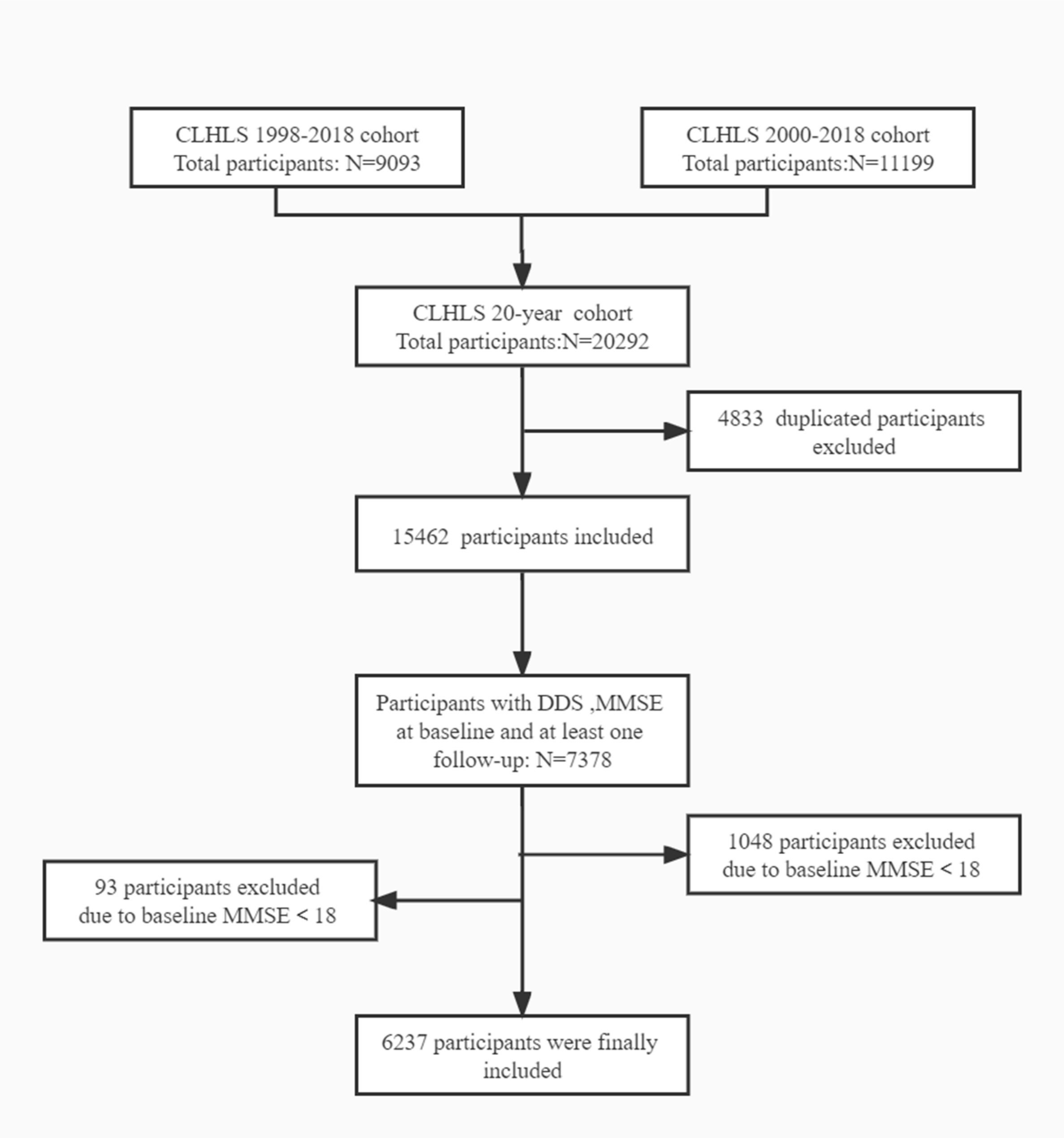

Table S1. The Chinese Version of the Mini-Mental State Exam (MMSE)[1].

Domains	MMSE items	Score (Total =30)
Orientation	What time of day is it right now (morning, afternoon, evening)?	1
	What is the animal year of this year?	1
	What is the date (day and month) of the mid-autumn festival?	1
	What is the season right now?	1
	What is the name of this county or district?	1
Naming	Please name as many kinds of food as possible in 1 minute (1 point for each food and 7 points for those who name 7 or more foods)	7
Registration	Table, apple, cloth. Please repeat these three objects.	3
Attention and calculation	I will ask you to spend $3 from $20, then you must spend $3 from the number you arrived at and continue to spend $3 until you are asked to stop.	5
Memory	Name the three objects learned earlier (table, apple, and cloth).	3
Language and visuospatial abilities	The individual is asked to draw a figure of overlapping pentagons.	1
	Naming pen and watch	2
	Repeating the following sentence: "What you plant, what you will get."	1
	The individual is asked to follow the interviewer's instruction: "Take the paper using your right hand, fold it in the middle using both hands, and place the paper on the floor."	3

Table S2. The association between DDS change patterns and risk of cognitive impairment in subgroups (univariate model).

Subgroups			DDS change patterns				DDS change score
	Events	Participants	High-High	High-Low	Low-High	Low-Low	
**Age(years)**							
80--89	857	3698	Reference	1.96 (1.59,2.43) ‡	1.38 (1.11,1.71) ‡	2.16 (1.80,2.60) ‡	0.97 (0.95,0.99) ‡
≥90	972	2539	Reference	1.38(1.12,1.69) ‡	1.15 (0.93,1.42)	1.69(1.42,2.01)‡	0.96(0.95,0.98) ‡
**Gender**							
Male	613	2893	Reference	1.68(1.32,2.13) ‡	1.23 (0.96,1.57)	1.99 (1.62,2.44) ‡	0.96(0.94,0.99) ‡
Female	1216	3344	Reference	1.56 (1.29,1.89) ‡	1.18 (0.97,1.43)	1.77(1.51,2.08) ‡	0.96(0.95,0.98) ‡
**Education**							
Illiterate	1309	3680	Reference	1.55(1.19,2.02) ‡	1.09(0.82,1.45)	1.87(1.48,2.36) ‡	0.96(0.93,0.98) ‡
Literate	520	2557	Reference	1.62 (1.35,1.94) ‡	1.22 (1.02,1.47)	1.78(1.53,2.07)	0.97(0.95,0.98) ‡
**Residence**							
Urban	738	3050	Reference	1.51(1.21,1.89) ‡	1.18(0.94,1.47)	2.16 (1.80,2.60) ‡	0.97(0.95,0.99) ‡
Rural	1091	3187	Reference	1.66 (1.35,2.03) ‡	1.26(1.02,1.55)	1.71(1.43,2.04) ‡	0.97(0.95,0.98) ‡
**Smoking status**							
Current or former smoker	545	2304	Reference	1.29 (0.99,1.68) ‡	1.08 (0.82,1.41)	1.43 (1.13,1.81) ‡	0.97(0.94,0.99) ‡
Non-smoker	1284	3933	Reference	1.71(1.43,2.05) ‡	1.25 (1.04,1.50)	1.90 (1.63,2.21) ‡	0.96(0.95,0.98) ‡
**Drinking**							

**status**							
Current or former drinker	591	2232	Reference	1.90 (1.46,2.46) ‡	1.54 (1.19,1.99)	2.53(2.04,3.15) ‡	0.97(0.94,0.99) ‡
Non-drinker	1238	4005	Reference	1.58(1.32,1.89) ‡	1.16(0. 96,1.39)	1.79 (1.54,2.09) ‡	0.96(0.95,0.98) ‡
**Regular exercise**							
Yes	540	2370	Reference	1.63 (1.28,2.08) ‡	1.18(0.91,1.54)	2.03(1.64,2.52) ‡	0.95(0.93,0.97) ‡
No	1289	3867	Reference	1.63 (1.35,1.96) ‡	1.19(0.99,1.44)	1.79(1.52,2.10) ‡	0.97(0.95,0.98) ‡
**Living pattern**							
Living with family	1505	5084	Reference	1.68 (1.43,1.97) ‡	1.24(1.05,1.46) ‡	2.03 (1.77,2.33) ‡	0.96(0.95,0.98) ‡
Living alone	324	1153	Reference	1.91(1.28,2.86) ‡	1.60(1.09,2.35) ‡	2.22(1.59,3.09) ‡	0.98(0.95,1.01)

‡ $p < 0.05$

Table S3. β-Coefficients and 95%CI for the association of the DDS change patterns with MMSE score changes over Follow-up Time (N=6237): results from Linear Mixed-effects models

DDS change model	MMSE score -β	95%CI	*P*-value
**Animal**-DDS change model			
**Baseline**			
High-high	**Reference**		
High-low	-0.270	-0.711， 0.096	0.172
Low-high	0.132	-0.329， 0.427	0.476
Low-low	-0.248	-0.660， -0.002	0.122
**Longitudinal**			
High-high×time	Reference		
High-low×time	-0.070	-0.122,0.051	0.244
Low-high×time	-0.141	-0.174,-0.014	0.010
Low-low×time	-0.089	-0.159,-0.021	0.060
**Plant**-DDS change model			
**Baseline**			
High-high	**Reference**		
High-low	-0.300	-0.656,0.0563	0.099
Low-high	0.166	-0.182,0.514	0.350
Low-low	-0.338	-0.643,-0.033	0.030
**Longitudinal**			
High-high×time	**Reference**		
High-low×time	-0.145	-0.253,-0.037	0.009
Low-high×time	-0.124	-0.225,-0.023	0.016
Low-low×time	-0.141	-0.230, -0.051	0.002

Table S4. Characteristics of 6237 participants at baseline

Variables	Total	Large loss	Small loss	Normal	Small gain	Large gain
No. of participants (%)	6237(100.0)	589(9.4)	1280 (20.5)	2268(36.4)	1483 (23.8)	617 (9.9)
Age, years, mean (SD)	88.55(6.93)	88.76(6.88)	88.89(7.07)	88.59(7.07)	88.37(6.79)	87.94(6.42)
**Age group**						
80-89	3698	341	741	1350	882	384
⩾90	2539	67	248	918	601	233
**Gender**						
Male	2893	259	608	1046	695	285
Female	3344	330	672	1222	788	332
**Type of residence**						
Urban	3050	269	594	1115	747	325
Rural	3187	320	686	1153	736	292
**Marital status**						
In marriage	1495	139	301	533	372	150
Not in marriage	4742	450	979	1735	1111	467
**Educational background**						
Illiteracy	2266	207	470	842	528	219
Literacy	3971	382	810	1426	955	398
**Living pattern**						
With family members	5084	486	1047	1850	1210	491
Alone or at nursing home	1153	103	233	418	273	126
**Tobacco smoking status**						
Non-smoker	3933	382	798	1434	931	388
Current smoker	1261	127	276	450	295	113
Former smoker	1043	80	206	384	257	116
**Drinking status**						
Non-drinker	4005	367	795	1485	936	422
Current drinker	1549	167	337	553	381	111
Former drinker	683	55	148	230	166	84
**Regular exercise**						
Yes	2370	227	520	877	546	200
No	3867	362	760	1391	937	417
**Number of teeth, mean (SD)**	6325	7.07 (11.06)	6.82 (10.21)	7.47 (12.64)	8.00 (12.88)	7.19 (11.39)
**Use of artificial denture**						
No	4556	421	967	1666	1058	444
Yes	1681	168	313	602	425	173
BMI, mean (SD), kg/m^2	6325	20.24 (4.41)	19.97 (4.32)	20.00 (4.28)	19.90 (4.25)	19.98 (4.45)
**Hypertension**						
No	5339	495	1098	1923	1297	526
Yes	898	94	182	345	186	91
**Diabetes**						
Yes	62	5	14	18	14	11
No	6175	584	1266	2250	1469	606
**Hear disease**						

No	5799	535	1183	2114	1398	569
Yes	438	54	97	154	85	48
**Cerebrovascular disease**						
No	6183	573	1253	2225	1451	601
Yes	142	16	27	43	32	16
**Digestive disease**						
No	6020	563	1237	2192	1431	597
Yes	217	26	43	76	52	20
**Cancer**						
No	6364	584	1277	2260	1479	617
Yes	20	5	3	8	4	0
**Respiratory disease**						
No	5591	527	1138	2003	1316	531
Yes	722	62	142	265	167	86
**Eye diseases**						
Yes	894	99	194	350	177	74
No	5343	490	1086	1918	1306	543
Baseline MMSE score, mean (SD)	26.32 (3.3)	26.39 (3.2)	26.48 (3.2)	26.32 (3.3)	26.25 (3.3)	26.05 (3.4)
Disability in ADL (activities of daily living), mean (SD)	5.67 (0.90)	5.58 (1.03)	5.66 (0.91)	5.67 (0.89)	5.69 (0.86)	5.68 (0.87)

*Data were expressed as counts (percentages), except for age and duration of follow-up; Missing data: 3 for occupation, 82 for BMI; 12 for education, 1 for smoke; 4 for drink; 55 for missing teeth;125 for diabetes; 109 for hypertension;121 for stroke and CVD;

Table S5. Subgroup analysis for the association of DDS change with cognitive impairment

DDS change	Events	Participants	HR (95%CI)	*P*-value
**Age (years)**				
**80--89**		3698		
Large decline	95	341	1.72 (1.34,2.21)	0.000
Small decline	198	741	1.46 (1.21,1.76)	0.000
Stable status	288	1350	Reference	
Small improvement	184	882	0.82 (0.68,0.99)	0.042
Large improvement	92	384	0.90 (0.70,1.15)	0.383
**≥90**		2539		
Large decline	121	248	1.68 (1.35,2.09)	0.000
Small decline	204	539	1.08 (0.91,1.29)	0.388
Stable status	363	918	Reference	
Small improvement	213	601	0.80 (0.68,0.96)	0.013
Large improvement	71	233	0.59 (0.45,0.77)	0.000
**Gender**				
**Male**		2893		
Large decline	78	260	1.79 (1.35,2.37)	0.000
Small decline	130	616	1.14 (0.91,1.43)	0.267
Stable status	217	1069	Reference	
Small improvement	140	709	0.81 (0.65,1.01)	0.057
Large improvement	54	291	0.75 (0.55,1.03)	0.073
**Female**		3344		
Large decline	437	1235	1.65 (1.35,2.03)	0.000
Small decline	140	334	1.28 (1.09,1.49)	0.002
Stable status	276	680	Reference	
Small improvement	261	795	0.83 (0.71,0.98)	0.025
Large improvement	110	336	0.77 (0.61,0.96)	0.018
**Education**				
**Illiterate**		3680		
Large decline	148	359	1.70 (1.39,2.08)	0.000
Small decline	301	743	1.37 (1.18,1.59)	0.000

Stable status	454	1327	Reference	
Small improvement	286	878	0.85 (0.73,0.99)	0.035
Large improvement	120	373	0.78 (0.63,0.96)	0.021
**Literate**		2557		
Large decline	59	208	1.67 (1.24,2.25)	0.001
Small decline	91	479	0.91 (0.71,1.17)	0.456
Stable status	174	866	Reference	
Small improvement	96	541	0.74 (0.58,0.94)	0.014
Large improvement	39	224	0.72 (0.51,1.03)	0.069

6.S-Sensitivity analyses

6.1 Excluded participants who lost to follow-up or died in the second 3 years

Table S6.1.1. The association between DDS change patterns and cognitive impairment

DDS change patterns	DDS change patterns from baseline to first follow up						
	High-high	High-low		Low-high		Low-low	
		**HR (95%CI)**	***P*-value**	**HR (95%CI)**	***P* -value**	**HR (95%CI)**	***P* -value**
Total DDS							
Model 1	Reference	1.45 (1.17,1.79)	0.001	1.11 (0.90,1.37)	0.336	1.55 (1.29,1.86)	0.000
Model 2‡	Reference	1.33 (1.07,1.65)	0.011	0.92 (0.74,1.14)	0.427	1.22 (1.01,1.48)	0.039

Model1*: Adjusted for age, sex;

Model2‡: Adjusted for model1 plus residence, education background, occupation, current marital status, living arrangement, tobacco smoking status, drinking status, regular exercise, number of teeth (continuous), use of artificial dentures, hypertension, diabetes, cerebrovascular diseases, respiratory diseases, digestive system diseases, ADL score, cancer, eye diseases and BMI (continuous), hear diseases. Other chronic diseases, baseline MMSE

Table S6.1.2. The association between DDS change and the incidence of cognitive impairment

	**Model1***		**Model2‡**	
	**HR (95%CI)**	***P*-value**	**HR (95%CI)**	***P*-value**
**DDS change (continuous)**	0.98 (0.96,1.00)	0.066	0.95 (0.93,0.98)	0.000
**Plant-based DDS**	0.98 (0.95,1.01)	0.174	0.95 (0.92,0.98)	0.001
**Animal-based DDS**	0.97 (0.93,1.01)	0.104	0.95 (0.91,0.99)	0.019
**DDS(categorical)**				
Large decline	1.38 (1.08,1.77)	0.010	1.58 (1.21,2.06)	0.001
Small decline	1.27 (1.06,1.52)	0.011	1.33 (1.10,1.61)	0.004
Stable status	Reference	Reference	Reference	Reference
Small improvement	1.08 (0.90,1.29)	0.405	0.99 (0.82,1.19)	0.885
Large improvement	1.19 (0.94,1.51)	0.151	0.93 (0.72,1.20)	0.559

Model 1*: Adjusted for age, sex;

Model2‡: Adjusted for model1 plus residence, education background, occupation, current marital status, living arrangement, tobacco smoking status, drinking status, regular exercise, number of teeth(continuous), use of artificial dentures, hypertension, diabetes, cerebrovascular diseases, respiratory diseases, digestive system diseases, ADL score, cancer, eye diseases and BMI(continuous), hear diseases. Other chronic diseases, baseline MMSE, baseline DDS

6.2 Using the definition of cognitive impairment (MMSE score less than 24)

Table S6.2.1. The association between DDS change patterns and cognitive impairment

DDS change patterns	DDS change patterns from baseline to first follow up						
	High-high	High-low		Low-high		Low-low	
		**HR (95%CI)**	***P*-value**	**HR (95%CI)**	***P*-value**	**HR (95%CI)**	***P*-value**
Total DDS							
Model 1	Reference	1.38 (1.24,1.53)	0.000	1.18 (1.07,1.33	0.001	1.46 (1.33,1.60)	0.000
Model 2‡	Reference	1.29 (1.15,1.43)	0.000	1.05 (0.94,1.17)	0.386	1.24 (1.12,1.36)	0.000

Model1: Adjusted for age and sex.

Model2: Adjusted for model1 plus residence, education background, occupation, current marital status, living arrangement, tobacco smoking status, drinking status, regular exercise, number of teeth(continuous), use of artificial dentures, hypertension, diabetes, cerebrovascular diseases, respiratory diseases, digestive system diseases, ADL score, cancer, eye diseases and BMI(continuous), hear diseases. Other chronic diseases, baseline MMSE

Table S6.2.2. The association between DDS change and the incidence of cognitive impairment

	**Model1**		**Model2**	
	**HR (95%CI)**	***P*-value**	**HR (95%CI)**	***P*-value**
**DDS change (continuous)**	0.99 (0.98,0.99)	0.003	0.96 (0.95,0.97)	0.000
**Plant-based DDS**	0.98 (0.97,0.97)	0.010	0.95 (0.94,0.97)	0.000
**Animal-based DDS**	0.98 (0.96,0.99)	0.025	0.96 (0.94,0.98)	0.000
**DDS(categorical)**				
Large decline	1.24 (1.10,1.40)	0.000	1.45 (1.28,1.65)	0.000
Small decline	1.05 (0.96,1.15)	0.300	1.13 (1.02,1.24)	0.015
Stable status	Reference	Reference	Reference	Reference
Small improvement	0.97 (0.89,1.07)	0.577	0.90 (0.82,0.99)	0.032
Large improvement	1.00 (0.89,1.14)	0.975	0.83 (0.73,0.95)	0.005

Model1: Adjusted for age and sex.

Model2: Adjusted for model1 plus residence, education background, occupation, current marital status, living arrangement, tobacco smoking status, drinking status, regular exercise, number of teeth(continuous), use of artificial dentures, hypertension, diabetes, heart diseases, cerebrovascular diseases, respiratory diseases, digestive system diseases, ADL score, cancer, eye diseases and BMI(continuous), hear diseases. Other chronic diseases, baseline MMSE, baseline DDS

6.3 Excluded participants with chronic diseases (hypertension, diabetes, cancer, cerebrovascular diseases) at baseline

Table S6.3.1. The association between DDS change patterns and cognitive impairment

DDS change patterns	DDS change patterns from baseline to first follow up						
	High-high	High-low		Low-high		Low-low	
		**HR (95%CI)**	***P*-value**	**HR (95%CI)**	***P*-value**	**HR (95%CI)**	***P*-value**
Total DDS							
Model 1	Reference	1.50 (1.27,1.77)	0.000	1.19 (1.01,1.40)	0.042	1.64 (1.42,1.89)	0.000
Model 2‡	Reference	1.38 (1.16,1.63)	0.000	1.02 (0.86,1.21)	0.805	1.35(1.16,1.56)	0.000

Model1: Adjusted for age and sex.

Model2: Adjusted for model1 plus residence, education background, occupation, current marital status, living arrangement, tobacco smoking status, drinking status, regular exercise, number of teeth(continuous), use of artificial dentures, hypertension, diabetes, cerebrovascular diseases, respiratory diseases, digestive system diseases, ADL score, cancer, eye diseases and BMI(continuous), hear diseases. Other chronic diseases, baseline MMSE

Table S6.3.2. The association between DDS change and the incidence of cognitive impairment

	**Model1**		**Model2**	
	**HR (95%CI)**	***P*-value**	**HR (95%CI)**	***P*-value**
**DDS change (continuous)**	0.97 (0.95,0.98)	0.000	0.93 (0.92,0.95)	0.000
**Plant-based DDS**	0.96 (0.94,0.98)	0.000	0.92 (0.90,0.94)	0.000
**Animal-based DDS**	0.96 (0.94,0.99)	0.014	0.94 (0.91,0.97)	0.000
**DDS (categorical)**				
Large decline	1.47 (1.24,1.74)	0.000	1.81 (1.51,2.16)	0.000
Small decline	1.08 (0.94,1.24)	0.290	1.20 (1.04,1.39)	0.014
Stable status	Reference	Reference	Reference	Reference
Small improvement	0.92(0.80,1.06)	0.245	0.86 (0.74,0.99)	0.037
Large improvement	1.01 (0.83,1.22)	0.928	0.79 (0.65,0.97)	0.025

Model1: Adjusted for age and sex.

Model2: Adjusted for model1 plus residence, education background, occupation, current marital status, living arrangement, tobacco smoking status, drinking status, regular exercise, number of teeth(continuous), use of artificial dentures, hypertension, diabetes, cerebrovascular diseases, respiratory diseases, digestive system diseases, ADL score, cancer, eye diseases and BMI (continuous), hear diseases Other chronic diseases, baseline MMSE, baseline DDS

6.4 Education was treated as a continuous variable

Table S6.4.1. The association between DDS change patterns and cognitive impairment

DDS change patterns	DDS change patterns from baseline to first follow up						
	High-high	High-low		Low-high		Low-low	
		**HR (95%CI)**	***P*-value**	**HR (95%CI)**	***P*-value**	**HR (95%CI)**	***P*-value**
Total DDS							
Model 1	Reference	1.56 (1.35,1.81)	0.000	1.18 (1.01,1.38)	0.028	1.70 (1.50,1.93)	0.000
Model 2‡	Reference	1.44 (1.24,1.68)	0.000	1.03 (0.88,1.20)	0.719	1.43 (1.26,1.64)	0.000

Model1: Adjusted for age and sex.

Model2: Adjusted for model1 plus residence, education background, occupation, current marital status, living arrangement, tobacco smoking status, drinking status, regular exercise, number of teeth(continuous), use of artificial dentures, hypertension, diabetes, cerebrovascular diseases, respiratory diseases, digestive system diseases, ADL score, cancer, eye diseases and BMI (continuous), hear diseases. Other chronic diseases, baseline MMSE

Table S6.4.2. The association between DDS change and the incidence of cognitive impairment

	**Model1**		**Model2**	
	**HR (95%CI)**	***P*-value**	**HR (95%CI)**	***P*-value**
**DDS change (continuous)**	0.97 (0.96,0.98)	0.000	0.93 (0.92,0.95)	0.000
**Plant-based DDS**	0.96 (0.95,0.98)	0.000	0.93 (0.91,0.95)	0.000
**Animal-based DDS**	0.95 (0.93,0.98)	0.000	0.92 (0.90,0.95)	0.000
**DDS (categorical)**				
Large decline	1.39 (1.20,1.63)	0.000	1.70 (1.44,2.01)	0.000
Small decline	1.11 (0.98,1.26)	0.100	1.23 (1.08,1.40)	0.001
Stable status	Reference	Reference	Reference	Reference
Small improvement	0.91 (0.80,1.03)	0.125	0.82 (0.73,0.94)	0.003
Large improvement	0.97 (0.82,1.15)	0.721	0.75 (0.63,0.90)	0.002

Model1: Adjusted for age and sex.

Model2: Adjusted for model1 plus residence, education background, occupation, current marital status, living arrangement, tobacco smoking status, drinking status, regular exercise, number of teeth(continuous), use of artificial dentures, hypertension, diabetes, cerebrovascular diseases, respiratory diseases, digestive system diseases, ADL score, cancer, eye diseases and BMI(continuous), hear diseases. Other chronic diseases, baseline MMSE, baseline DDS

Reference

1. Liu, M.; He, P.; Zhou, C.; Zhang, Z.; Zhang, Y.; Li, H.; Ye, Z.; Wu, Q.; Yang, S.; Zhang, Y., et al. Association of waist-calf circumference ratio with incident cognitive impairment in older adults. *Am J Clin Nutr* **2022**, *115*, 1005-1012, doi:10.1093/ajcn/nqac011.
